# High amino acid diversity and positive selection at a putative coral immunity gene (*tachylectin-2*)

**DOI:** 10.1186/1471-2148-10-150

**Published:** 2010-05-19

**Authors:** Marshall L Hayes, Ron I Eytan, Michael E Hellberg

**Affiliations:** 1 Department of Plant Pathology and Plant-Microbe Biology, Cornell University, Ithaca, NY 14853, USA; 2 Department of Biological Sciences, Louisiana State University, Baton Rouge, LA 70803, USA

## Abstract

**Background:**

Genes involved in immune functions, including pathogen recognition and the activation of innate defense pathways, are among the most genetically variable known, and the proteins that they encode are often characterized by high rates of amino acid substitutions, a hallmark of positive selection. The high levels of variation characteristic of immunity genes make them useful tools for conservation genetics. To date, highly variable immunity genes have yet to be found in corals, keystone organisms of the world's most diverse marine ecosystem, the coral reef. Here, we examine variation in and selection on a putative innate immunity gene from *Oculina*, a coral genus previously used as a model for studies of coral disease and bleaching.

**Results:**

In a survey of 244 *Oculina *alleles, we find high nonsynonymous variation and a signature of positive selection, consistent with a putative role in immunity. Using computational protein structure prediction, we generate a structural model of the *Oculina *protein that closely matches the known structure of tachylectin-2 from the Japanese horseshoe crab (*Tachypleus tridentatus*), a protein with demonstrated function in microbial recognition and agglutination. We also demonstrate that at least three other genera of anthozoan cnidarians (*Acropora, Montastrea *and *Nematostella*) possess proteins structurally similar to tachylectin-2.

**Conclusions:**

Taken together, the evidence of high amino acid diversity, positive selection and structural correspondence to the horseshoe crab tachylectin-2 suggests that this protein is 1) part of *Oculina's *innate immunity repertoire, and 2) evolving adaptively, possibly under selective pressure from coral-associated microorganisms. *Tachylectin-2 *may serve as a candidate locus to screen coral populations for their capacity to respond adaptively to future environmental change.

## Background

Host immune systems must be able to recognize a wide range of rapidly evolving microbes; thus, functional variation is a hallmark of responses to potential pathogens and other non-self molecules. Such variation can arise via complex interactions leading to somatic recombination, as in vertebrate immune systems and molluscan fibrinogen-related proteins [1], or more simply via genetic diversity in the host immune system, either at the level of families of genes or alleles at a single locus. Genes used by potential hosts to distinguish self from non-self and to recognize and defend against pathogenic microbes include some of the most genetically variable known, among them those encoding the Major Histocompatibility Complex (MHC) proteins in vertebrates [2] and a histocompatibility protein in tunicates [3], as well as disease resistance (*R*) proteins [4] and ribonucleases of gametophytic self-incompatibility (GSI) [5] in plants.

High levels of variation are generated by diversifying selection that results from either selection favoring heterozygotes or from frequency-dependent selection favoring rare alleles. A molecular signature of positive selection (that is, an excess in the nonsynonymous nucleotide substitution rate dN relative to the synonymous rate dS when compared to neutral expectations) is therefore another distinctive feature of host immunity genes. Indeed, one of the first uses of the now-standard dN/dS ratio approach for detecting positive selection was on sequence data from the MHC binding cleft of mice and humans [6]. Subsequent work on natural populations of vertebrates has detected positive selection at MHC many times [7], as well as on other immunity genes from vertebrates [8] and plants [9,10].

Because high variation at immunity genes appears to be sustained by selection, surveys of intraspecific variation at such loci have been employed in ways that neutral markers cannot be. For example, the signature of positive selection at MHC may be difficult to maintain if historical population sizes were small enough for drift to dominate, a situation that can be evaluated by comparing patterns at MHC loci and highly variable, but presumably neutral, nuclear markers such as microsatellites [11]. More generally, surveys of variation at MHC loci have taken on a special role in a conservation context, where they have been seen as proxies for levels of standing adaptive variation in wild populations [12,13]. Such assessments of a population's ability to withstand challenges from pathogens should hold true to the extent that variation at immunity genes relates to an organism's fitness and ability to fend off pathogens and parasites, as has been seen for MHC in some vertebrates [14-16].

In contrast to the many works on MHC variation in wild vertebrate populations, findings of immunity gene variation in invertebrate animals are only beginning to emerge. In part, such surveys have been stymied by the heterogeneous ways in which the immune systems of different phyla work [17]. Schulenburg *et al. *[18] also suggested that invertebrate immunity genes may be limited in their genetic variability, but recent studies suggest otherwise. Exceptionally high diversity has been recorded recently in invertebrate immunity genes, both among paralogous members of the same gene family (*e.g. *in sea urchins [19]; in nematodes: [20]) and among alleles at a single locus (*e.g. *mosquitoes: [21]; in mussels: [22]). High polymorphism has not been accompanied universally by positive selection however [23], although most studies have not tested for it.

Corals (Phylum Cnidaria, Class Anthozoa, Order Scleractinia) are among the many taxa for which we have no information on the population-level variability of immunity genes. Reef-building (hermatypic) corals are especially suitable targets for studies of immunity gene variation for several reasons. Reef corals create habitat that sustains large numbers of other species, yet these corals have been in decline globally in the recent past [24]. Coincident with this decline has been a rise in reported coral diseases [25,26]. These have often been associated with high water temperature anomalies (*e.g. *[27]), although there are claims for mechanistic ties between coral bleaching and bacteria [28,29]. The long generation times of many large hermatypic corals would seem to make them especially vulnerable to rapidly evolving microbes with generation times perhaps 10^7^-10^8 ^shorter. The search for polymorphic recognition genes from corals that could effectively match the diversity of potential pathogens is just beginning. One carbohydrate recognition protein (Millectin) from a coral binds both bacterial pathogens and algal symbionts [30], but of the several similar isoforms reported, all came from a single individual, suggesting that they constituted a recently radiating gene family and were non-allelic. A polymorphic kin-recognition locus has been characterized in a hydroid [31], but no such highly variable region has been reported from corals, nor has any coral immunity gene been characterized for intraspecific variation to date.

Here, we characterize sequence variation at a putative coral immunity gene, *tachylectin-2*, fortuitously identified while generating markers from an EST library [32] from *Oculina*, a genus that has served previously as a model for studies of coral disease and bleaching [28]. Tachylectin-2 was originally isolated from the Japanese horseshoe crab (*Tachypleus tridentatus*) and has since been demonstrated experimentally to possess anti-microbial activity [33]. Its crystal structure is unique and composed of a five-bladed β-propeller [34], each blade of which potentially binds N-acetyl sugars such as those associated with lipopolysaccharide (LPS) and peptidoglycan found in the bacterial cell wall.

Homologs of tachylectin-2 have been reported from two cnidarians, although in both cases, functional roles other than host immunity have been proposed. Mali *et al. *[35] isolated a gene (*CTRN*) from the hydrozoan *Hydractinia echinata *that had a primary sequence with a similarity of over 30% to horseshoe crab tachylectin-1 and a typical tachylectin repeat structure. However CTRN was expressed solely in circumoral neurons and, most importantly, expression levels were not induced when challenged with LPS. This suggests that the primary function of CTRN is neither host immunity nor some other form of microbial recognition. Schwarz *et al. *[36] identified a homolog of horseshoe crab tachylectin-2 via BLASTX screening of an EST library generated from the coral *Montastrea faveolata*. They speculated that such a protein could mediate interactions between coral hosts and their algal symbionts and suggested further study to investigate this possibility.

To confirm the identity of the *Oculina *gene, we show that the inferred structure of the *Oculina *tachylectin-2 corresponds closely to the solved crystal structure of the horseshoe crab tachylectin-2. We also report high nonsynonymous variation for the *Oculina tachylectin-2 *and demonstrate that positive selection has promoted allelic diversity at this locus. Our results are consistent with adaptive diversification at a host immunity gene, and thus open the door to molecular studies of host susceptibility and population vulnerability in corals.

## Results

### Cnidarian Homologs of Tachylectin-2 from *Tachypleus tridentatus*

**Figure 1:**
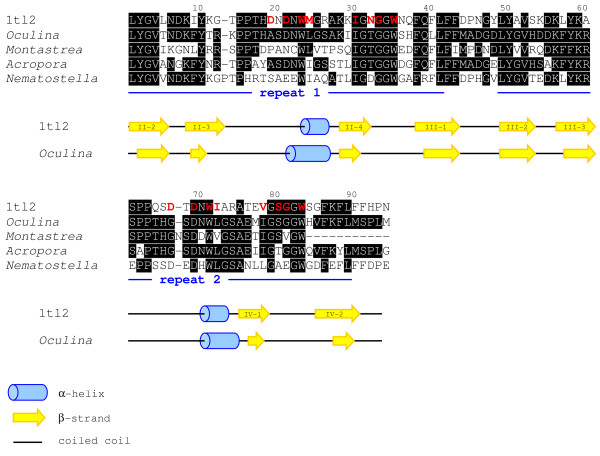
**Amino acid sequence alignment of conserved regions of the horseshoe crab tachylectin-2 (1tl2), and tachylectin-2s from the anthozoans *Oculina*, *Montastrea*, *Acropora *and *Nematostella***. Black boxing indicates conserved sites, relative to the *Oculina *sequence. Red text highlights those positions in 1tl2 that are functional N-acetyl-sugar-binding sites [34]. The locations of the 40-amino acid tandem repeats characteristic of tachylectin-2 are indicated by the blue lines. Below the sequences, the predicted secondary structural elements of the *Oculina *tachylectin are shown relative to elements of the experimentally derived secondary structure of 1tl2.

Pairwise alignments of putative tachylectin-2 amino acid sequences from four cnidarians (three corals and an anemone) and the full-length tachylectin-2 from the Japanese horseshoe crab reveal a high degree of similarity among the proteins, beginning at position 54 of the horseshoe crab tachylectin-2 (Figure 1, Table 1). The secondary structure prediction corroborates the localization of our alignment and indicates that the *Oculina *sequence corresponds to a region spanning nearly two complete tandem repeats in the horseshoe crab tachylectin-2, including two β-strands within β-sheet II, all four strands of β-sheet III and the first two strands of β-sheet IV with each β-sheet being separated by a single α-helix (Figure 1, [34]). Furthermore, consistent with the characteristics of a 5-bladed β-propellor, the *Oculina *primary structure is comprised of highly similar (70% identity) tandem repeats of 40 amino acids each (**LYGV**XX**DKFY**X**R**X**PPTH**X**SDNWLGSA**XX**IG**X**GGW**XX**F**XX**L**, Figure 1). Within each of its five equivalent β-sheets, the horseshoe crab tachylectin-2 harbors an individual N-acetyl sugar-binding site comprised of 8 functional residues capable of direct interaction with target ligands. The partial *Oculina *sequence may thus contain 16 binding residues, including a complete 8-residue binding pocket. Moreover, these 16 binding residues are highly conserved between the two proteins. Ten residues are identical to the horseshoe crab tachylectin-2 sequence, while 3 are conserved substitutions (M23L, I70L and V76I), and 3 are semi-conserved (D18A, N31T and D65G).

**Table 1:** Anthozoan tachylectin-like sequences and their similarities to tachylectin-2 from *Tachypleus tridentatus*

Host species	Accession Number	sequence length (aa)	% identity/similarity to *Oculina *sequence	% identity/similarity to 1tl2 aa 54-140
*Oculina varicosa*	FJ966784	92	100/100	60/68
*Montastrea faveolata*^*a*^	FE038913	111	54/60	44/62
*Acropora millepora*^*b*^	EZ038328	277	76/85	52/64
*Nematostella vectensis*^*c*^	EDO38290	242	52/67	55/71

^*a *^Local alignment of *M. faveolata *is with 1tl2 aa 54-135.

^*b *^Local alignment of *A. millepora *with 1tl2 aa 54-140 is presented here, although the strongest alignment is with 1tl2 aa 7-95.

^*c *^Local alignment of *N. vectensis *is with 1tl2 aa 54-145.

**Figure 2:**
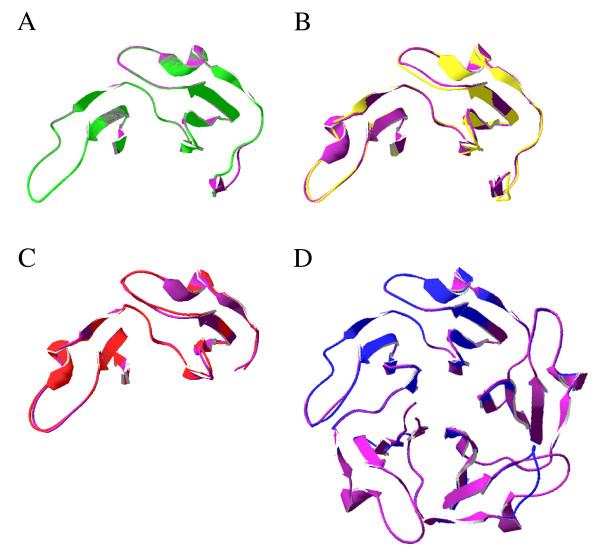
**Ribbon diagrams of tachylectin-2s from *Oculina *(A), *Acropora (B)*, *Montastrea *(C) and *Nematostella *(D)**. Each structural model has been superimposed on the known structure of 1tl2 (purple), with the *Oculina *structure represented in green, *Acropora *in yellow, *Montastrea *in red and *Nematostella *in blue.

As an additional test of structural correspondence, we generated three-dimensional (3-D) structural models for the *Oculina*, *Acropora, Montastrea *and *Nematostella *homologs and used a threading approach to align the models with the known X-ray crystal structure for tachylectin-2 [PDB:1tl2] from horseshoe crab. For each of the four models, the estimated precision, or percent likelihood of a correct threading match based on e-values, is 100%. Furthermore, respective RMS deviations resulting from the superposition of the *Oculina*, *Acropora, Montastrea and Nematostella *models onto 1tl2 are 0.42 Å (on 372 atoms), 0.67 Å (on 352 atoms), 0.65 Å (on 324 atoms) and 0.41 Å (on 896 atoms) (Figure 2). Very similar, well-aligned structures generally have RMS deviations <1.0 Å, indicating that all four of our alignments are precise. Divergence among these models and 1tl2 is minimal and limited to the C-terminal extensions in the particular cases of *Oculina *and *Acropora*. Thus, a phylogenetically wide range of corals possesses tachylectin-2-like proteins that are very similar to and align well with the horseshoe crab tachylectin-2.

### Positive Selection

**Figure 3:**
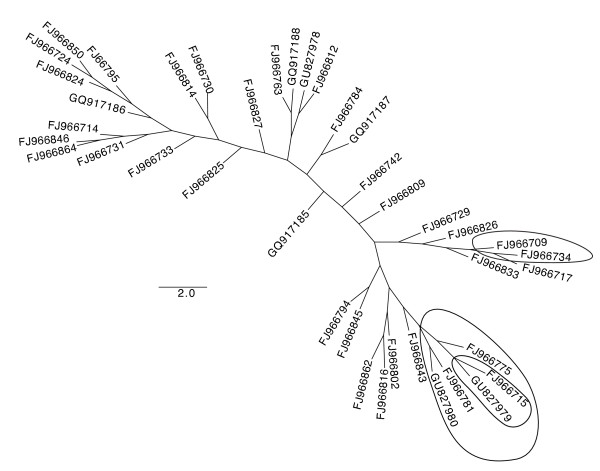
**Maximum likelihood tree for tachylectin-2 alleles**. Unrooted maximum likelihood tree depicting relationships between alleles used in the analyses to detect positive selection. The three circled groups have >50% bootstrap support in an alternative Neighbor Joining tree. Abundance and geographical distribution of alleles are given in Table 4.

Diversity at *tachylectin-2 *as indicated by **π **(the number of nucleotide differences per site between two randomly chosen sequences) was high overall (**π = **0.0179), and was evident at both silent (π_Syn _= 0.0299) and replacement sites (π_Rep _= 0.0145). Waterson's theta (**θ**_**W**_) was 0.0215. The maximum likelihood analysis recovered a single best tree (Figure 3), although support for nodes was low (as expected for intraspecific data from species lacking strong phylogeographic structure). The neighbor joining tree (Figure 3) supported just three nodes with bootstrap values >50%, and thus was essentially a star phylogeny.

Likelihood ratio tests suggest that positive selection promotes nonsynonymous substitutions among our sample of *tachylectin-2 *alleles from *Oculina *(Table 2). The strength of this conclusion varies depending on which allele tree the tests are based upon. Both tests (M7 vs. M8 and M1a vs. M2a) based on the collapsed neighbor joining (cNJ) tree were highly significant. These tests test indicated that while only a small proportion of residues were under selection (about 5.5%), those residues were under very strong selection, with the ratio of nonsynonymous to synonymous substitution rates (ω) > 9. Based on the single best maximum likelihood tree, the M7 (where ω can take on a range of values between 0 and 1) versus M8 (as for M7, but with an additional positive selection category where ω can exceed one) test was significant. The test comparing M1a (with just two ω categories: neutrality (ω = 1) and another between 0 and 1) to M2a (as for M1a, but with an additional positive selection category where ω > 1) was not significant (p = 0.081). For these tests, a higher proportion (about 24%) of codons were inferred to fall in the class experiencing positive selection, but the ω for this class was not as high as for the collapsed NJ analysis (ω = 2.37).

**Table 2:** Tests for positive selection on putative *tachylectin-2 *from *Oculina *based on maximum likelihood (ML) and collapsed Neighbor Joining (cNJ) allele trees

Models compared (allele tree)	2ΔL	P1	ω
M1a vs. M2a (ML)	5.02	23.9	2.37

M7 vs. M8 (ML)	6.18*	23.9	2.37

M1a vs. M2a (cNJ)	95.2**	5.6	9.38

M7 vs. M8 (cNJ)	96.8**	5.7	9.36

* - significant at p = 0.05

** - significant at p < 0.001

p1 - percentage of sites under positive selection (under model M8)

ω - ratio of dN/dS for those sites under positive selection (model M8)

**Figure 4:**
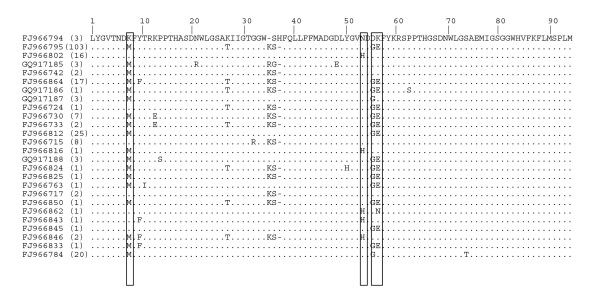
**Amino acid alignment of *tachylectin-2 *alleles from *Oculina varicosa***. Values in parentheses are the number of times that an allele was found in a geographic survey of variation; populations where each allele was found are in Table 4. Four boxed residues are under positive selection based on Bayesian posterior probabilities of ≥ 90% using model M8 based on two alternative allele trees. Additional variable positions are under positive selection based on BEB posterior probabilities of ≥50% using the best ML tree (residues 10, 11, 13, 14, 21, 48 and 71) and a collapsed NJ tree (residue 27).

Consistent with the estimated proportions of residues under selection (Table 2), the BEB analysis for sites under positive selection (Table 3) based on the cNJ tree indicated fewer sites (just 5) under stronger selection than the analysis based on the maximum likelihood tree (which indicated 11 residues). One of the sites that was significant for the analysis based on the cNJ tree was not significant for the analysis based on the ML tree. Thus, a total of 12 residues were flagged as potentially evolving under positive selection (Figure 4).

**Table 3:** *Oculina tachylectin-2 *codons under positive selection

	Probability (ω > 1)	
Residue #	ML	cNJ

8	0.91	1.0

10	0.92	

11	0.54	

13	0.84	

14	0.55	

21	0.69	

27		1.0

48	0.52	

51	0.90	0.97

53	0.90	1.0

54	0.98	1.0

71	0.53	

The *p*-values for the M7 vs. M8 likelihood ratio tests are shown, as are the sites determined to be under positive selection by Bayes Empirical Bayes analysis based on two allele trees (maximum likelihood, ML, and collapsed neighbor joining, cNJ) and their probabilities of belonging to the group of residues undergoing positive selection.

An in-frame indel occurs in the *Oculina *alignment. The alternate resolution of the alignment without the indel results in five consecutive nucleotide substitutions and two non-synonymous replacements (data not shown). The in-frame indel used in our alignment, by eliminating non-synonymous substitutions, results in a more conservative test for positive selection by removing two sites from consideration in the PAML analyses.

Tajima's D (-0.680) and Fu and Li's D (-2.17) were both negative, consistent with an excess of low frequency polymorphisms, but these values were not statistically significant. Fay and Wu's H (0.631) was also not significant.

Tests for recombination generally did not reveal significant results. The DSS test implemented in TOPALi did not detect recombination in the *tachylectin-2 *alignment. Two tests for recombination were implemented using the Datamonkey server, the first looking for a single recombination event (SBP test) and the second for multiple recombination events (GARD test). Neither test detected any recombination. The four-gamete test detected six recombination events; the estimate of *R *per gene was 128.0.

### Spatial and Functional Correspondence of Positively Selected Sites in *Oculina *Tachylectin-2

**Figure 5:**
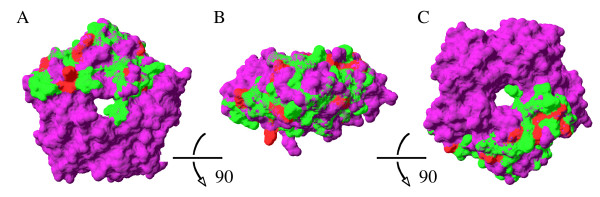
**Molecular surface diagrams of the *Oculina *tachylectin-2**. Top (A), side (B) and bottom (C) views of the structural model of the *Oculina *tachylectin-2 (green and red shading) reveal the spatial coverage of the partial *Oculina *sequence when superimposed on the known structure of 1tl2 (purple). Successive views are achieved by rotating the molecule downward 90° on a horizontal plane. Residues shaded in red are those *Oculina *sites that have been identified as being under positive selection, as determined by BEB posterior probabilities of ≥50%.

The results of the Bayes Empirical Bayes (BEB) analysis (Table 3, Figure 4) indicated that 12 codons in *Oculina *tachylectin-2 may have been subject to positive selection. To determine whether the spatial organization of these sites corresponded to regions of functional importance, positively selected sites were first mapped onto the threaded structural model. In the space-filling representation of the modeled molecular surface (Figure 5), all 12 positively selected sites are localized on the protein's exterior and are well exposed. This observation is independently confirmed by calculations of solvent-accessible surface area using the GETAREA algorithm and 10% solvent accessibility as a cut-off value.

Next, we tested whether the spatial distribution of positive selection was clustered, or otherwise nonrandom, in 3-D space by comparing observed codon positions to random permutations of an equal number of surfaces sites along the same length of protein. No significant trend in selected site clustering was observed. Indeed, selected sites appear to occur along the entire length of β-strands, α-helices and coiled coils present in the *Oculina *tachylectin-2 (*cf. *Figure 2, Figure 4).

Finally, we examined whether the 12 positively selected sites correspond to active N-acetyl sugar-binding residues or are otherwise directly associated with the known functional regions of the horseshoe crab tachylectin-2. Based on our sequence alignment and the degree of conservation at known functional positions in 1tl2, no positively selected site corresponded to any active site within an N-acetyl sugar-binding pocket.

## Discussion

### A Recognition Function for Cnidarian Tachylectin-2 Homologs

Since the initial discovery of tachylectins in the Japanese horseshoe crab *Tachypleus tridentatus*, tachylectin-like proteins have been identified in a wide range of organisms including slime molds [37], sponges [38], hydrozoan cnidarians [35], lancelets [39] and fish [40,41]. This coverage has been recently extended to anthozoan cnidarians on the basis of genome sequence from a sea anemone [42] and EST sequences from three corals [32,36,37]. Thus, tachylectin-2-like proteins are present in at least two cnidarian classes (Hydrozoa and Anthozoa) as well as in both major clades of scleractinians, the complex (*e.g. Acropora*) and the robust (*e.g. Montastrea*, *Oculina*) [43].

Proteins of the tachylectin family have broad-spectrum anti-microbial activity [44] and play a role in non-self recognition [45], two functions central to cnidarian innate immunity [46,47]. Alignments and structural modeling of translated amino-acid sequences show that our *Oculina *gene encodes a protein homologous to tachylectin-2. Although our characterization of the *Oculina tachylectin-2 *is limited because we have yet to identify the start site for the gene's coding region, several lines of circumstantial evidence suggest that the partial *Oculina *gene encodes an ortholog of *tachylectin-2*. First, based on pairwise sequence comparisons (Figure 1) and protein threading results (Figure 2), the sequences from *Oculina *and *Tachypleus *are highly similar. Second, partial amino-acid sequences from *Oculina *reveal an internal homology that consists of two highly similar tandem repeats, a feature characteristic of protein regions that arrange pseudo-symmetrically to form larger structures comprised of multiple β-propeller folds. Lectins, and tachylectins among them, characteristically derive conformational rigidity, multivalency, and avidity from these β-propeller structures [48,49]. Finally, the predicted secondary structure (*i.e. *α-helices, β-strands and coiled coils) of the *Oculina *protein corresponds closely to the experimentally derived X-ray structure of 1tl2 (Figure 1).

Modeling the structure of *Oculina *tachylectin-2 enables us to map the location of residues found to be under positive selection on the 3-D structure of the protein. Without exception, replacements have occurred at well-exposed sites on the protein's surface. This external localization most likely stems from surface residues having lower structural constraints than their buried counterparts (*e.g. *[50,51]). Thus, positive selection does not appear to have altered the structure and stability of the core of *Oculina *tachylectin-2, nor has selection targeted residues known to be involved in carbohydrate recognition and binding. These have been conserved, suggesting that the molecular affinity of the *Oculina *tachylectin-2 for N-acetyl sugars remains unchanged. However, given that positive selection acts on residues on the surface of the *Oculina *protein, especially those at the fringes of the N-acetyl sugar-binding pockets (*cf. *Figure 5 here and Figure 5 from [34]), these selected changes may modulate the architecture, flexibility and specificity of the binding pocket to accommodate a broader range of ligand sizes and configurations from a more diverse pool of non-self sources. Such correlations between structure and activity have been previously described for other classes of lectin (*e.g. *for galectins, [52]).

### Positive Selection on *Oculina Tachylectin-2*

In our survey of *Oculina tachylectin-2 *alleles, we found high nonsynonymous variation (25 alleles differing by amino replacements in a survey of 244 alleles) promoted by positive selection (Table 3, Figure 4). Because high variation and positive selection are disproportionately observed in immunity genes relative to other functional gene classes [53], the presence of these signatures is consistent with *Oculina tachylectin-2 *performing some aspect of coral immunity (even though some immunity genes do not reveal such a signal, e.g. [54]). Experimental assessments of proposed immune functions are critical both to confirming the involvement of this protein in non-self recognition or some other form of coral-microbe interaction based on N-acetyl sugar specificity, and to identifying the selective forces driving the evolution of tachylectin-2.

An alternate explanation to a purely immune explanation for positive selection on tachylectin-2 is that the protein may mediate a more general process of non-self recognition, limited not only to pathogen detection but also including symbiont identification. Indeed, in the best-characterized host-symbiont interactions (that between legumes and rhizobia), De Mita *et al. *[55,56] found positive selection in the legume *Medicago trunculata *at the *NODULATION RECEPTOR KINASE *(*NORK*) gene, which functions during the early stages of root infection by symbiotic nitrogen-fixing bacteria and endomycorrhizal fungi. While the authors propose that *Medicago*-rhizobium interactions may co-evolve in a manner reminiscent of host-pathogen co-evolution, this interpretation is a matter of current debate [57]. The signature of positive selection has also appeared in a study trying to elucidate the molecular underpinnings of specificity among corals and their dinoflagellate symbionts in terms of lectin-mediated interactions. Voolstra *et al. *[58] presented preliminary evidence of positive selection at a dinoflagellate gene encoding a novel protein of unknown function although, unlike our study, this conclusion of positive selection was not based on allelic variation. Until more studies conclude that adaptive evolution acts to diversify alleles at host symbiont recognition genes, the positive selection seen among alleles at *Oculina tachylectin-2 *should be considered more consistent with a role in immunity than symbiosis.

In our earlier work on several nominal *Oculina *species [32], we employed three nuclear loci (*tachylectin-2*, as well as two others tentatively identified using BLASTP as fatty acid elongase and elongation factor 1α) as markers to reveal subdivision among 10 different sampling populations spanning a 2370 km range and also to establish the genetic isolation of a threatened deep-water population (Jeff's Reef on the Oculina Banks off central Florida). In the latter case, the *tachylectin-2 *locus was unusual both in being fixed for a single allele and in being putatively associated with host immunity. This observation adds to the ongoing debate over the adaptive potential of reef building corals in the face of global climatic change [59,60]. On the one hand, most *Oculina *populations segregate ample genetic variation and show the signature of positive, diversifying selection at the *tachylectin-2 *gene, demonstrating that genotypic diversity and adaptive variation are present. On the other, the deep-water population, presently threatened by illegal trawling [61], is fixed for a single *tachylectin-2 *allele, which may indicate its future prospects are even bleaker than demography suggests.

That functional genetic variation for host resistance exists within coral populations has been shown recently for another Atlantic species, *Acropora cervicornis*. Vollmer and Kline [62] showed that there are differences in resistance to white-band disease resulting from *in-situ *transmission assays among different *A. cervicornis *genotypes. Genotypes immune to white-band disease occurred at low frequency (6%). This may indicate diversifying selection, as strong directional selection acting on genes involved in the immune response might have been expected to sweep a disease resistant genotype to fixation during the mass die-off of *A. cervicornis *due to white-band disease. Such diversifying selection would be consistent with our observations in the *tachylectin-2 *gene in *Oculina*.

The geographic variation in allelic diversity and positive selection in our data are also intriguing in light of recent studies demonstrating that allelic fixation may provide a signature of geographically variable selection in isolated populations [63]. For example, in a geographical survey of variation at 16 immunity genes in six natural populations of teosinte, Moeller and Tiffin [64] found a marked difference in allelic variation at a single locus, the wound-inducible serine protease inhibitor (*wip1*), in a single population. Taken along with other observations (for example, this population was fixed for a replacement at an active site), *wip1 *appeared to have undergone a population-specific selective sweep. The results of Moeller and Tiffin also suggest that such signatures are relatively rare, even among immunity genes. Considering the special import in variation at immunity genes in evaluating population viability for conservation purposes [13], it would be interesting to explore patterns of interpopulation variation in *Oculina *or other scleractinians using a more comprehensive set of candidate loci including both immunity and non-immunity genes.

Given that our inference of positive selection was based on allelic sequences, the possible role of recombination in creating this signal deserves consideration. Tests for recombination were equivocal. On one hand, the results of the four-gamete test [32] and values for *R *indicate multiple recombination events and a high per-locus recombination rate. This rate (128) is high enough to cause false positives in site-based tests of selection, especially the M7 vs. M8 test [65]. On the other hand, values for *R *have been shown to be biased upwards [65,66] and are sensitive to infinite-site violations, which are present in our dataset. Further, neither the DSS test nor the GARD test detected multiple recombination events, even though both are designed to do so. Even if we allow for the presence of one recombination event, this does not appear to be a high enough rate of recombination to cause false positives in the site-based tests of selection [67,68]. The four-gamete test determines whether all four combinations of a pair of variable sites are present in a sample [69]. If so, then recombination is inferred. However, incompatible sites can arise through recurrent mutations as well as recombination. For data sets where the mutational process is best described by any time-reversible mutation model (as ours is), incompatible sites will arise, causing the four-gamete test to be overly conservative (in that it may flag recombination even when it is not present). Indeed, one tachylectin-2 site was dropped from the four-gamete test because it segregates for more than two nucleotides, reinforcing the argument that an infinite sites model is not appropriate for our data.

## Conclusion

The combination of two lines of evidence suggests the gene region examined here is involved in coral innate immunity. First, both primary amino acid sequence and inferred protein structure are similar to those of tachylectin-2, a protein from horseshoe crabs associated with an innate immune response to bacterial pathogens. Second, the divergence of alleles at *tachylectin-2 *has been promoted by positive selection, a hallmark of immunity genes. This proposed immunity function should be confirmed experimentally. Nonetheless, given the conservation threats faced by many corals and the special role of genetic variation at immunity genes such as the vertebrate MHC in assessing population viability, the gene encoding tachylectin-2 in corals may serve as a candidate locus to screen anthozoan populations for their potential to respond adaptively to future challenges. Neither widespread assessments of immunity loci nor comparisons of immunity to non-immunity genes have been conducted for corals as they have for other hosts (*e.g. *[63,70-72]). Combining the identification of additional coral immunity genes with geographic surveys and detailed studies of the functional consequences of naturally occurring variation should provide insights into both how corals defend themselves against natural enemies and how better we can preserve these key components of marine biodiversity.

## Methods

### Protein Alignments, Threading, and Structural Analysis of Positively Selected Sites

A *tachylectin-2*-like sequence was first identified during random sequencing of an *Oculina varicosa *cDNA library [32]. A BLASTX survey of GenBank, using the *Oculina *sequence as a query, identified a putative tachylectin from the larval transcriptome of the reef-building coral, *Acropora millepora *[GenBank:EZ038328][73]. A *Montastrea faveolata *homolog [FE038913], previously described by Schwarz *et al. *[36] in an EST screen, was retrieved from GenBank. Finally, a *Nematostella vectensis *homolog [EDO38290] was located in the JGI genome database http://genome.jgi-psf.org/Nemve1/Nemve1.home.html using conserved motifs from the *Oculina *sequence as a BLASTX query. Protein sequences were aligned using MegAlign (DNASTAR) for multiple alignments and EMBOSS http://www.ebi.ac.uk/Tools/emboss/align/index.html for pairwise alignments.

Three-dimensional (3-D) structures of tachylectin-2 based on amino acid sequences from *Oculina*, *Acropora, Montastrea *and *Nematostella *were modeled using computational tools for structure prediction available in the PHYRE Protein Homology/analogY Recognition Engine version 0.2 ([74]; http://www.sbg.bio.ic.ac.uk/phyre/). The PHYRE protocol incorporates four discrete processing steps into one program interface: 1) generation of a protein profile from the user-provided sequence using iterative PSI-Blast to identify both close and remote sequence homologs, 2) prediction of secondary structure by three independent programs (Psi-Pred, SSPro and JNet), 3) prediction of ordered and disordered regions using Disopred, and 4) fold recognition via the application of a profile-profile alignment algorithm to a reference fold library [75]. The resulting product is a downloadable model with associated confidence estimates relating the model to existing structures in the Structural Classification of Proteins (SCOP) and Protein Data Bank (PDB) databases.

Models for tachylectin-2s from *Oculina*, *Acropora, Montastrea *and *Nematostella *were visualized and further manipulated using DEEPVIEW version 4.0.1 ([76]; http://spdbv.vital-it.ch/). The PDB file of the *T. tridentatus *tachylectin-2 [PDB:1tl2] was downloaded from the RCSB Protein Data Bank and similarly viewed in DEEPVIEW. Tachylectin-2 sequences from *Oculina*, *Acropora, Montastrea *and *Nematostella *were structurally superposed on 1tl2 using DEEPVIEW's Iterative Magic Fit function, and the resulting quality of fit was computed as a Root Mean Squared (RMS) deviation in angstroms. To determine the degree to which positively selection sites were either buried or on the protein's surface, solvent accessible surface area for individual residues of *Oculina *tachylectin-2 mapped onto 1tl2 was calculated using GETAREA version 1.0 beta ([77]; http://curie.utmb.edu/getarea.html). Finally, statistical significance of the spatial clustering of positively selected amino acids and correspondence with functional binding sites was assessed using programs from Clark and Swanson [78].

### Tests for Positive Selection

To test for positive selection acting on *Oculina tachylectin-2*, we assembled a dataset consisting of a subset of our population genetic data (Table 4) from Eytan *et al. *[32]. The 3' UTR was cut from the original alignment, yielding 40 non-identical alleles (each 276-bp in length), 25 of which differed by non-synonymous substitutions.

**Table 4:** Sequences used in this study

GenBank Acc. #	Frequency	Populations where present
FJ966794	3	NC

FJ966795	99	NC(21), GA(24), JAX(16), DAY(13), FtP(7), HSH(2), CFL(5), SAR(7), PAN(4)

FJ966802	16	NC(2), GA(2), DAY(4), SAR(8)

GQ917185	2	GA

FJ966742	2	GA

FJ966864	17	GA, FtP, HSH(2), SAR(2), PAN(11)

FJ966775	3	JAX

FJ966781	1	JAX

FJ966731	3	JAX, DAY, SAR

FJ966715	8	JAX, FtP(2), HSH(3), CFL, PAN

GQ917186	1	DAY

GQ917187	3	DAY(2), PAN

GU827980	1	DAY

FJ966724	1	DAY

FJ966729	2	DAY

FJ966733	2	DAY

FJ966730	7	DAY(2), FtP, HSH. SAR, PAN(2)

FJ966734	2	DAY, HSH

FJ966812	25	FtP(10), HSH(5), CFL(7), PAN(3)

GU827979	3	FtP, CFL, PAN

FJ966816	1	FtP

FJ966809	1	FtP

GQ917188	3	FtP, HSH, PAN

FJ966814	1	FtP

FJ966824	1	FtP

FJ966825	1	FtP

FJ966826	1	FtP

FJ966827	1	FtP

FJ966763	1	HSH

FJ966709	1	CFL

FJ966714	2	CFL

FJ966717	2	CFL

FJ966843	1	SAR

FJ966845	1	SAR

FJ966846	1	SAR

FJ966850	1	SAR

FJ966862	1	SAR

FJ966833	1	PAN

GU827978	1	PAN

*FJ966784	20	JR80

Total	244	

Frequency indicates the number of allele copies encountered over a geographic survey of 244 alleles from 10 different populations [32]. * indicates the fixed allele from the deep water (80 m) population at Jeff's Reef (JR80). Locality abbreviations: NC (North Carolina), GA (Georgia), JAX (Jacksonville, Florida), DAY (Daytona Beach, Florida), FtP (Fort Pierce, Florida), HSH (Horseshoe Reef, Florida), CFL (Cape Florida), SAR (Sarasota, Florida), PAN (Panama City, Florida)

CODEML, implemented in PAML v4.2 [79] was used to conduct tests for selection using the site models, which allow the omega ratio to vary among codon sites. Two pairs of null and alternate models were used that provide two likelihood ratio tests (LRTs) for positive selection. The first compares the null model of nearly neutral molecular evolution (the M1a model) to the alternate model of positive selection (M2a) [80]. The second compares a model of beta-distributed variable selection pressure (M7) to an alternate model of beta-distributed variable selection pressure plus positive selection (M8) [81]. The LRT employing two degrees of freedom was used to determine if positive selection was present. Codon sites under positive selection were determined using the Bayes Empirical Bayes analysis [82], which has been shown to be both powerful and not excessively prone to false positives [80,82].

A phylogenetic tree is required for the site-based tests of selection implemented in PAML. Before constructing the tree, a model of sequence evolution was determined with jModelTest v0.1.1 [83] using the AIC to choose between models. The TPM2+I model was selected as the appropriate model. A heuristic tree search using maximum likelihood was implemented in GARLI [84]. Starting trees were obtained via stepwise addition. 100 random-addition sequence replications were performed with TBR branch-swapping. Optimal trees from each repetition were saved. The best tree from all the replicates was then used for tests of selection in PAML. Although the ML tests for positive selection implemented by PAML are generally robust to tree topology [85], we also ran the analysis for an alternative Neighbor Joining allele tree (implemented in PAUP* v4b10 [86]) to insure any inference of positive selection were not overly dependent on a single topology.

We also tested for selection using summary statistic-based methods. Tajima's D, Fu and Li's D, and Fay and Wu's H were all implemented in DNAsp v5.0 [87]. To conduct Fay and Wu's H test, a single *tachylectin-2 *sequence from the coral *Solenastrea hyades *(GenBank: FJ966866) was used as an outgroup, and significance was tested using coalescent simulations (without recombination) as implemented in DNAsp. Summary statistics for levels of variation (π and θ_W_) were also calculated using DNAsp.

Recombination events can cause the failure of tree-based tests for detecting non-neutral evolution of codons [65], with high rates of false-positives being particularly problematic [67]. We tested for the presence of recombination in the tachlylectin-2 alignment using several different methods. The tests that we used were: the DSS method [88] implemented in TOPALi v2.5 [89], the SBP and GARD methods of Kosakovsky-Pond *et al. *[68] implemented online via the Datamonkey webserver [90], and the four-gamete test [91] implemented in DNAsp v5.0. The preferred [68] corrected AIC criterion was used to evaluate the significance of these tests. In addition, we calculated Hudson's *R*, the per-locus population scaled recombination rate [91], also implemented in DNAsp v5.0.

## Authors' contributions

All authors carried out the molecular genetic studies and drafted the manuscript. RIE performed the phylogenetic analyses and statistical analyses of variation and positive selection. MLH conducted the protein modeling and visualization. MEH conceived of the study and coordinated its design. All authors read, edited and approved the final manuscript.

## Authors' information

MLH applies molecular genetics, functional genomics and biochemical approaches to the study of host-microbe interactions, using plants, invertebrates and bacteria as model organisms. RIE is interested in the origin and maintenance of marine biodiversity, particularly in coral reef taxa. MEH is broadly interested in population isolation, speciation, and molecular evolution in marine animals, especially anthozoans and gastropods.
